# Investigation of CO_2_ Orientational Dynamics through Simulated NMR Line Shapes**

**DOI:** 10.1002/cphc.202100489

**Published:** 2021-09-23

**Authors:** Patrick Melix, Thomas Heine

**Affiliations:** ^1^ Universität Leipzig Wilhelm-Ostwald-Institut für Physikalische und Theoretische Chemie Linnéstraße 2 04103 Leipzig Germany; ^2^ Northwestern University Department of Chemical and Biological Engineering 2145 Sheridan Road Evanston Illinois 60208 United States; ^3^ TU Dresden, Professur für Theoretische Chemie Bergstr. 66c 01062 Dresden Germany; ^4^ Yonsei University Department of Chemistry Seodaemun-gu Seoul 120-749 Republic of Korea

**Keywords:** NMR, metal-organic frameworks, molecular dynamics, DUT-8, CO2, line shape

## Abstract

The dynamics of carbon dioxide in third generation (i. e., flexible) Metal‐Organic Frameworks (MOFs) can be experimentally observed by ^13^C NMR spectroscopy. The obtained line shapes directly correlate with the motion of the adsorbed CO_2_, which in turn are readily available from classical molecular dynamics (MD) simulations. In this article, we present our publicly available implementation of an algorithm to calculate NMR line shapes from MD trajectories in a matter of minutes on any current personal computer. We apply the methodology to study an effect observed experimentally when adsorbing CO_2_ in different samples of the pillared layer MOF Ni_2_(ndc)_2_(dabco) (ndc=2,6‐naphthalene‐dicarboxylate, dabco=1,4‐diazabicyclo‐[2.2.2]‐octane), also known as DUT‐8(Ni). In ^13^C NMR experiments of adsorbed CO_2_ in this MOF, small (rigid) crystals result in narrower NMR line shapes than larger (flexible) crystals. The reasons for the higher mobility of CO_2_ inside the smaller crystals is unknown. Our ligand field molecular mechanics simulations provide atomistic insight into the effects visible in NMR experiments with limited computational effort.

## Graphical abstract

Calculating ^13^C NMR line shapes from MD simulations is a cost‐effective way to gain insight into the dynamics of adsorbed CO_2_ using the experimentally observed line shape broadening. A simple to use Python library capable of calculating NMR line shapes from trajectories is presented and applied to systems of CO_2_ adsorbed in the pillared layer Metal‐Organic Framework DUT‐8(Ni), which exhibit specific NMR line shapes depending on the size of the framework crystals.

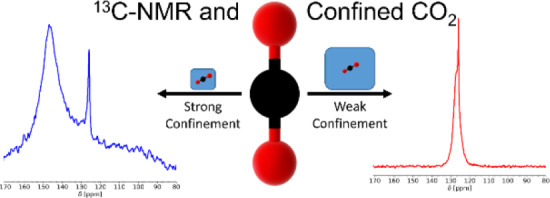

## Introduction

For the study of carbon dioxide in solid host systems, ^13^C NMR can be an immensely powerful tool.[^1^, ^2^, ^3^, ^4^] The chemical shift (δ ) of any nucleus per se depends, besides on the structural environment within the molecular structure also on the orientation of the molecule with respect to the external magnetic field. In ideal solutions, this orientational dependence is averaged out ($\bar{\delta }$ ) due to rapid movement of the species under investigation. This is the reason for the characteristically narrow lines observed in solution NMR experiments. In contrast, this averaging of the chemical shift does not occur in solid state NMR experiments. In these, the species' movement is mostly slower and therefore the anisotropy of the measured signal is retained. The experimental NMR line shapes are therefore directly related to the molecular motion of the CO_2_ molecules in the sample. In simple terms: the more movement the CO_2_ molecules exhibit, the narrower the measured line will be. For an overview of the methodology we refer the interested reader to an excellent review by Reimer *et al*.^[2]^ Commonly, the experimental line shapes are used to fit chemical shift (or shielding) tensors, which can be used to obtain relative positional and motional information about the CO_2_ molecules.[^5^, ^6^, ^7^] The opposite route can however also be taken: from molecular dynamics (MD) simulations, the trajectories of the adsorbed CO_2_ molecules are readily available. This information can be translated into a residual chemical shift anisotropy (i. e. a chemical shift line width) following two (very similar) previously published methodologies,[^8^, ^9^] one of which we implemented in a publicly available Python package.^[10]^ This simulation‐based approach is neither limited to a specific host system, nor to CO_2_ as the adsorbate.

**Figure 1:**
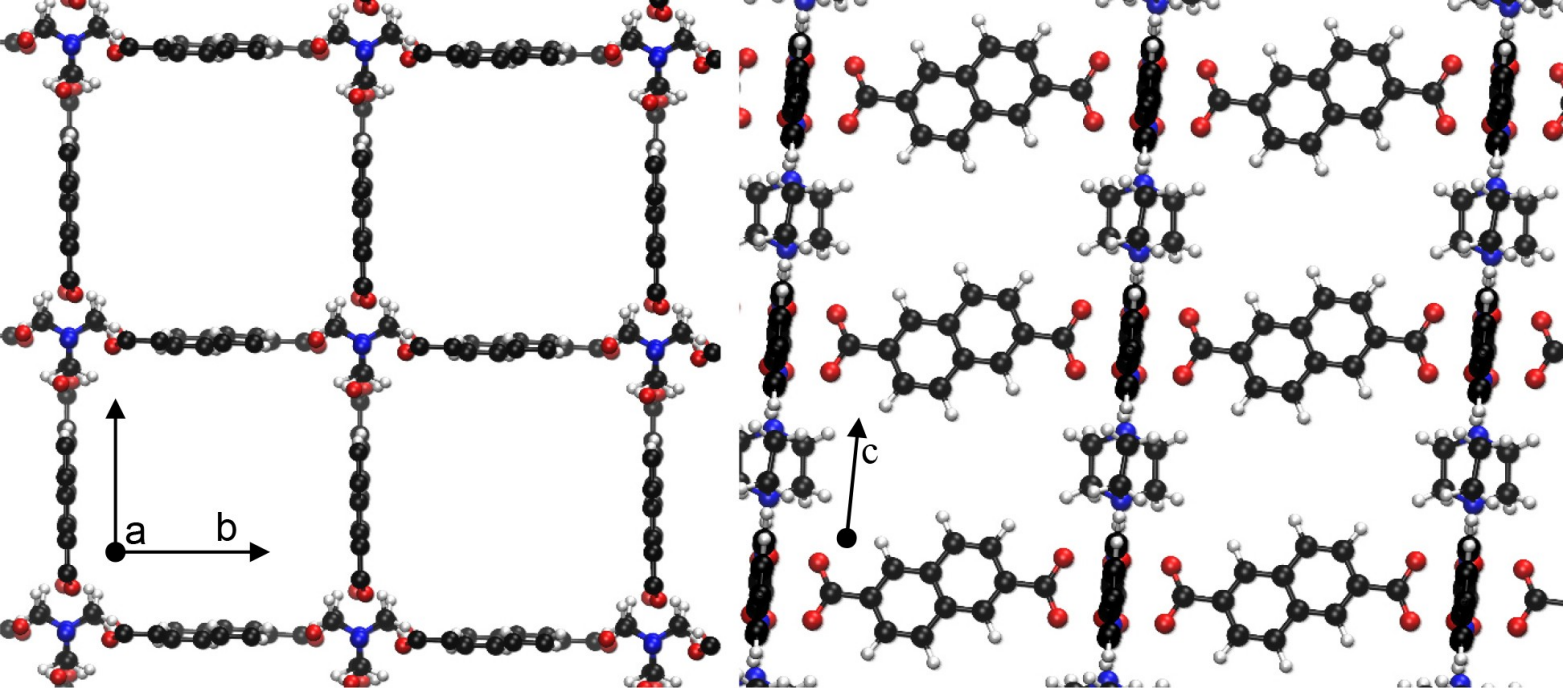
Graphical representation of DUT8‐(Ni) conformer **B** in its open phase (**op**), viewed along the pillar axis (left, c axis) and along ndc linkers (right, a or b axis). Atoms colored by element: Nickel ochre, oxygen red, nitrogen blue, carbon black and hydrogen white. Reproduced under the terms of the Creative Commons Attribution 4.0 License.^[41]^ Copyright 2020, Zenodo.

**Figure 2:**
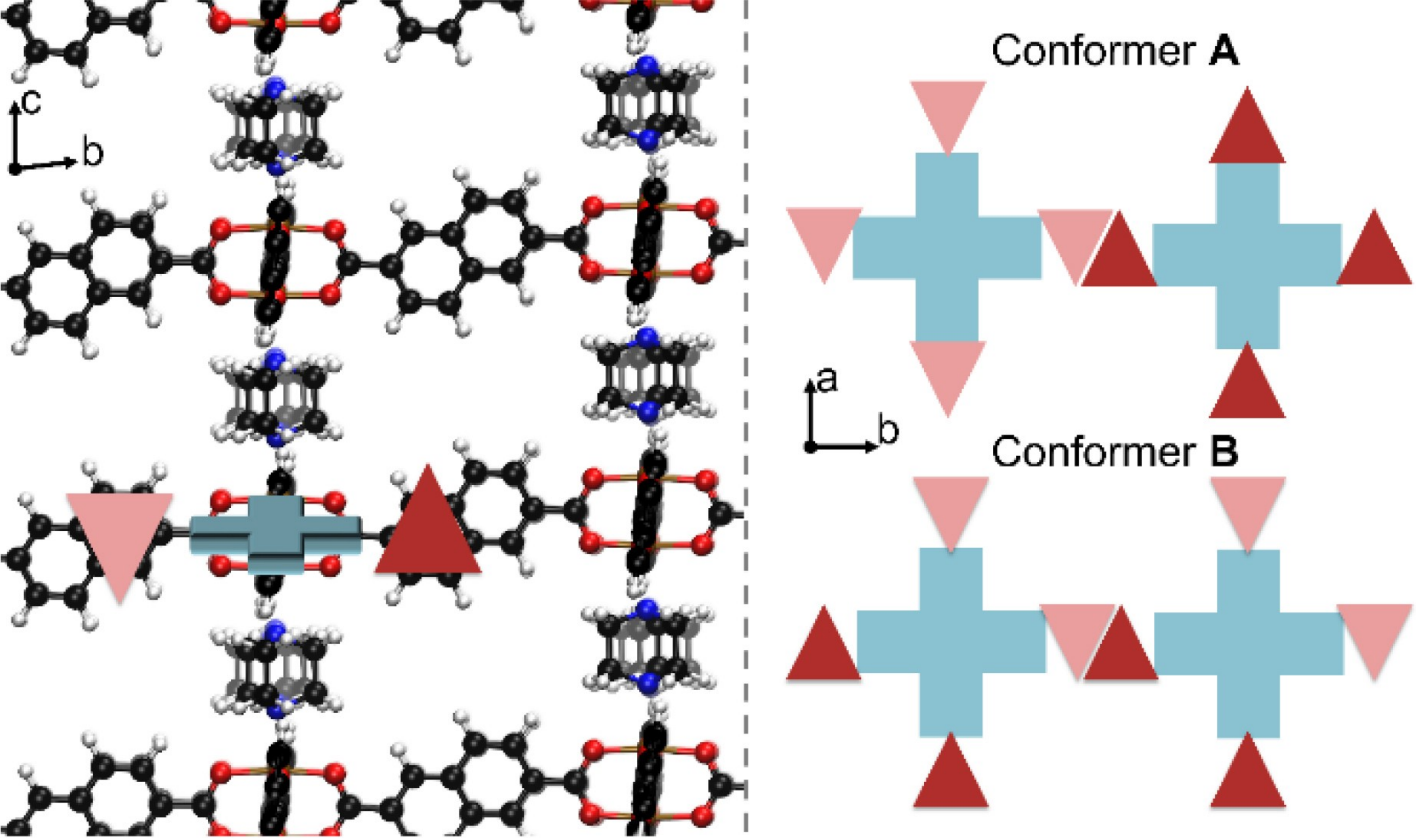
Graphical representation of DUT8‐(Ni) conformer **B** in its open phase viewed along the ndc linkers (left). Schematic representation of linker orientations in the **A** and **B** conformer (right). Triangles represent a half ndc linker, either pointing up (dark red) or down (light red) with respect to the closest metal node (blue crosses). Reproduced under the terms of the Creative Commons Attribution 4.0 License.^[40]^ Copyright 2021, Zenodo.

Breathing Metal‐Organic Frameworks (MOFs),[^11^, ^12^, ^13^, ^14^, ^15^, ^16^] especially in combination with carbon dioxide, have seen a lot of focus over the last years. Investigations into their applicability in e. g. gas sensing, separation and storage have been studied extensively due to the environmental implications of the topic. The pillared layer MOF DUT‐8(Ni)^[17]^ is one of the representatives of this class of materials. It consists of layers of Ni_2_(COO)_4_ paddle wheels connected by nonlinear ndc (2,6‐naphthalene‐dicarboxylate) linkers and stacked by pillaring layers with dabco (1,4‐diazabicyclo‐[2.2.2]‐octane). Figure 1 shows a top (along the Ni‐dabco‐Ni axis) and side view (perpendicular to one half of the ndc linkers) of the structure. One of its remarkable characteristics is the possibility to access two distinct phases by ad‐ or desorbing guests into/from the structure. After synthesis, the structure is in a solvent‐filled open pore state (denoted **op**). Upon solvent removal, it can collapse (unit cell volume changes to less than half the initial volume) into a closed pore state (denoted **cp**). This collapse is, however, not observed if the synthesized crystals are smaller than a certain threshold (approximately 500 nm in length).[^18^, ^19^, ^20^] The size dependence of the flexibility is not a unique characteristic of DUT‐8(Ni) and has been observed and studied in other materials.[^21^, ^22^] Another important factor for the flexibility of DUT‐8(Ni) is the exact conformation of the material. As shown previously, the nonlinearity of the ndc linkers give rise to conformational isomerism.^[23]^ We showed that certain conformers result in a specific stacking of the ndc linkers in the **cp** phase, increasing the dispersion interactions and therefore stabilizing it with respect to the **op** phase. Two relevant isomers were determined, an **A** conformer where all linkers of a paddle wheel point up or down, and a **B** conformer where opposing linkers on one paddlewheel point in opposite directions (see Figure 2). Conformer **A** was found to be “rigid” (i. e. not transforming into the **cp** phase) in contrast to the “flexible” (i. e. transformation into **cp** is possible) conformer **B**. Recently, a more realistic modelling using nanodomains of differing conformers lead to an even better understanding of the real crystal system.^[24]^ The reverse transition of opening the structure from its **cp** phase to the **op** phase can be achieved by adsorbing guest molecules.[^18^, ^20^, ^23^, ^25^, ^26^, ^27^, ^28^, ^29^, ^30^, ^31^, ^32^, ^33^] Under the range of guest molecules able to reopen flexible DUT‐8(Ni) are CO_2_ and CH_4_.

**Figure 3:**
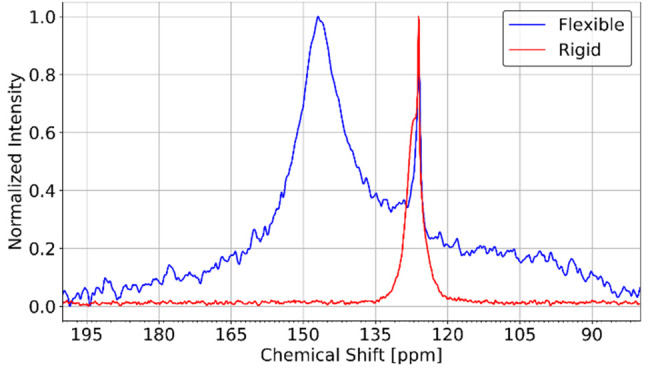
Measured ^13^C NMR spectra of CO_2_ adsorbed in flexible (blue) and rigid (red) samples of DUT‐8(Ni) at 215 K. Flexible sample measured at a partial CO_2_ pressure of 5.1 bar (2 : 1 CO_2_/CH_4_ mixture, no CH_4_ adsorption) and rigid sample measured at a CO_2_ pressure of 6.6 bar. Both samples are in the **op** state at these experimental conditions. Adapted with permission from Sin et al., Langmuir, 2019, 35 (8), 3162–3170. Copyright 2019 American Chemical Society.^[30]^

In a previous study of Sin *et al*.,^[30]^ the co‐adsorption of CO_2_ and CH_4_ in rigid and flexible crystals of DUT‐8(Ni) was studied by ^13^C NMR. The residual anisotropy (i. e. the line width of the NMR signal) of the ^13^C NMR signal of ^13^C labelled CO_2_ can be directly related to the dynamic orientation of the CO_2_ molecules.[^2^, ^8^] Sin *et al*. found that the residual chemical shift anisotropy of CO_2_ is significantly smaller in the submicron‐sized, rigid crystals than in the micrometer‐sized, flexible crystals (see Figure 3). The measured ^13^C signal is for both rigid and flexible crystals much broader than in gaseous CO_2_ (the gas peak is observed at approx. 126 ppm, see Figure 3), some dynamic order therefore remains even in the smallest crystals measured. This observation could back then not be attributed to a single cause, but two possible reasons were suggested: First, the smaller anisotropy could be caused by faster inter‐particle exchange. Or second, the flexible framework could apply an ordering force onto the adsorbed CO_2_ molecules that is not present in the rigid framework.^[30]^

Using a line shape simulation methodology that has been successfully applied to MOFs,^[8]^ we can distinguish between these two possibilities. From molecular dynamics (MD) simulations we extract the trajectories of the adsorbed CO_2_ molecules. We then calculate the residual chemical shift anisotropy (i. e. the chemical shift line width) based on these trajectories, following a previously published methodology^[8]^ that we implemented in a publicly available Python package.^[10]^ A similar methodology based on a different powder averaging algorithm was also published by Alavi *et al*. in 2008.^[9]^

## Experimental Section

### MD Simulations

We employ our previously published Ligand‐Field Molecular Mechanics (LFMM) simulation setup for DUT‐8(Ni).[^34^, ^35^] We perform three types of MD simulations: 1) CO_2_ in the rigid **A** conformer of DUT‐8(Ni) using a fully flexible cell (N**σ**T ensemble); 2) CO_2_ in the **B** conformer with a fully flexible cell (N**σ**T ensemble); 3) CO_2_ in the **B** conformer with a fixed cell (NVT ensemble, no restraints on atomic positions) in the **op** state. The simulation time of all simulations is 1 ns and temperatures of 200, 300 and 400 K are used for each of the three systems. For details of the LFMM parametrization see Ref. [34], further details of the MD simulations are given in Section 1.1 of the Supporting Information and adhere to the previous publications.[^34^, ^35^]

### Line shape Simulation

In order to simulate the residual chemical shift anisotropy, we follow a slightly altered version of the calculation procedure published in ref.^[8]^ The theoretical background is presented in more detail in Section 1.2.1 of the Supporting Information, as it is thus far only completely available in German.^[36]^ It can shortly be described by the following procedure: For an anisotropic, linear molecule like CO_2_, the shielding tensor **σ** contains two contributions, one for parallel ($\delta_{\left|\right|}$ ) and one for perpendicular ($\delta_{⊥}$ ) orientation to the external magnetic field $\vec{B_{0}}$ . Since the chemical shifts δ of CO_2_ for parallel and perpendicular orientation are known ($\delta_{\left|\right|}=$ −90 ppm and $\delta_{⊥}=$ 245 ppm, using TMS as Ref. [37]) and the chemical surrounding is anisotropic in only one spatial dimension, the chemical shift δ can be expressed as a function of only the orientation of the CO_2_ molecule with respect to the external magnetic field $\vec{B_{0}}$ .

The orientation of CO_2_ molecules is approximated by the vector between the oxygen atoms ($\vec{r}$ ) and stored along the trajectory. All vectors are summed into a tensor – using the formula:

$$\sum_{i=1}^{N}\frac{\vec{r_{i}}⊗\vec{r_{i}}}{\vec{r_{i}}\cdot \vec{r_{i}}},$$

where N represents the number of CO_2_ molecules and ⊗ denotes the dyadic product). This tensor represents the orientation of all molecules and can be calculated over any number of simulation steps. By diagonalizing this tensor and feeding it to a powder averaging algorithm (here the algorithm reported by Alderman *et al*.^[38]^ is used), relative line shapes can be calculated very efficiently (in a matter of minutes on any recent desktop or laptop computer). Spectra calculated by this procedure are with respect to a relative chemical shift Δ . This relative chemical shift Δ is equal to the chemical shift anisotropy (i. e. the line width of the NMR signal) of perfectly aligned, frozen CO_2_, which is known to be $\Delta =\delta_{⊥}-\delta_{\left|\right|}=$ 335 ppm.^[37]^ The linewidth of the calculated relative NMR signals can therefore directly be compared to the line width from experiment by multiplying it by Δ= 335 ppm. As mentioned above, this approach is only valid when the host has a neglectable influence on the guests shielding tensor.

Details regarding the implementation are given in Section 1.2.2 of the Supporting Information.

## Results and Discussion

**Figure 4:**
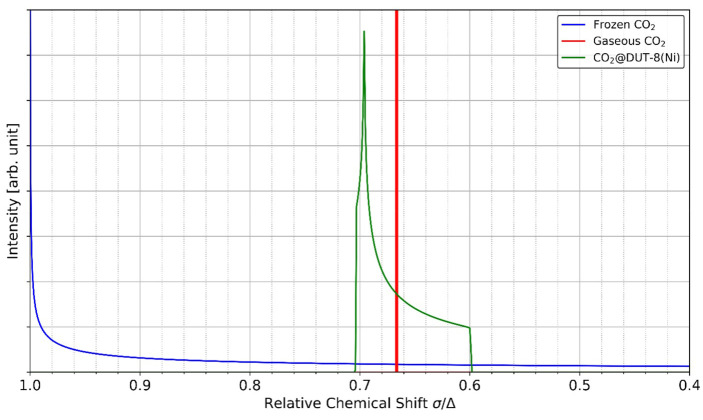
Calculated relative ^13^C NMR spectra for perfectly aligned, frozen CO_2_ (blue), gaseous CO_2_ (red) and an exemplary result from our simulations (green). The example given is the result from our rigid cell 400 K simulation with 18 molecules of CO_2_. Reproduced under the terms of the Creative Commons Attribution 4.0 License.^[40]^ Copyright 2021, Zenodo.

For the two extreme cases of gaseous CO_2_ (single peak of infinite height at $\bar{\delta }/\Delta =2/3$ , although a finite height and width arise from the numerical accuracy of the powder averaging algorithm) and perfectly aligned (i. e. frozen) CO_2_ (line shape follows a functional dependence of ${\left(4\sqrt{1-\bar{\delta }/\Delta }\right)}^{-1}$ ) the calculated relative ^13^C NMR spectra can be visualized as shown in Figure 4. Simulations of CO_2_ molecules exhibiting a partial ordering, like adsorbed CO_2_ in DUT‐8(Ni), yield a relative spectrum as the one given in Figure 4. The applicability of the method (a neglectable influence of the host on the shielding tensor of the guest) and the sampling have been validated as outlined in Section 1.2.3 and 1.2.4 of the Supporting Information. The relevant measure for comparison with experimental data is the width of the calculated spectra. In the example given in Figure 4, this amounts to approximately 0.1 which is equal to a residual shift anisotropy (i. e. a line width) of 33.5 ppm. To assert the validity of the observed line widths, we calculated the instantaneous temperature of the MOF, the guest molecules and the entire system based on the final atomic velocities. The resulting temperatures are given in Section 2.2 of the Supporting information. No significant deviation from the set thermostat temperature is observed.

**Figure 5:**
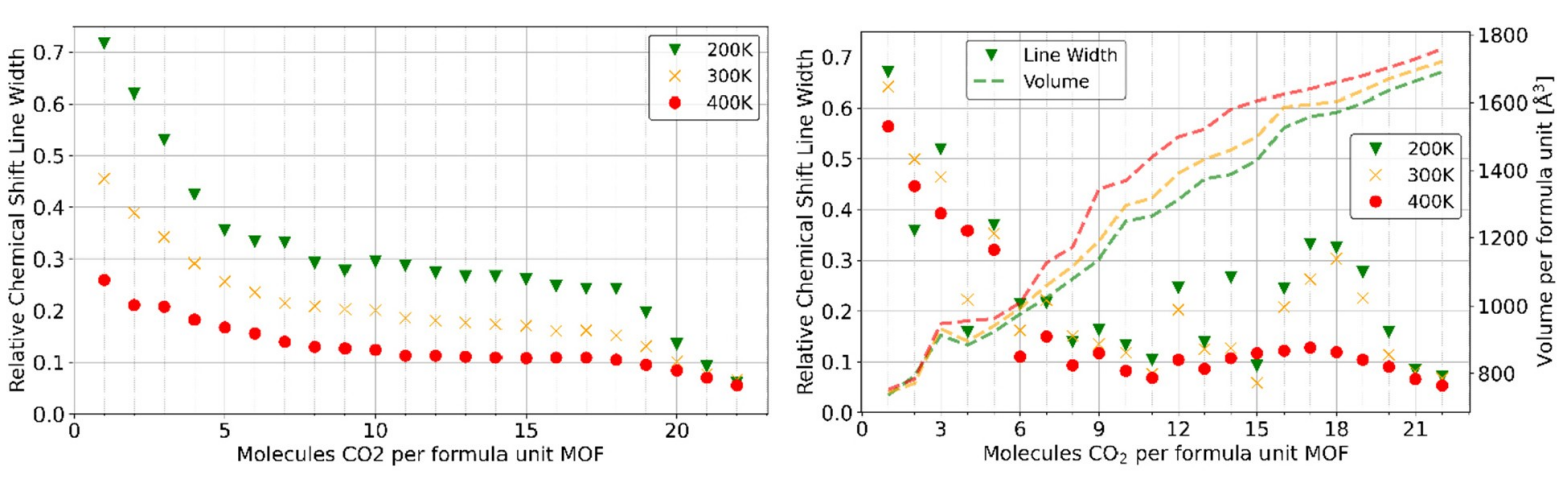
Relative chemical shift line widths with respect to the number of CO_2_ molecules loaded per formula unit MOF at 200 K (green), 300 K (yellow) and 400 K (red) simulation temperature. Left: rigid cell simulations of the **B** conformer in the **op** state. Right: Flexible cell simulations of **B** conformer, cell volumes given as dashed lines. Experimentally, the maximum loading is expected to be in the range of 18 to 22 molecules per formula unit MOF (see text). Reproduced under the terms of the Creative Commons Attribution 4.0 License.^[40]^ Copyright 2021, Zenodo.

The accumulated relative line widths of the rigid and flexible cell simulations with respect to the loading are given in Figure 5 (the corresponding plot of simulations of the **A** conformer are given in Figure S15 of the Supporting Information). All calculated line shapes are given in Sections 2.4 to 2.6 of the Supporting Information. Low loadings in the flexible simulation cell (right panel of Figure 5) exhibit a high ordering (large line width) across all temperatures, as the closed framework confines the few adsorbed CO_2_ molecules. In the rigid cell (left panel of Figure 5, same volume as the fully loaded flexible cell at the corresponding temperature), low loadings exhibit more CO_2_ movement than in the flexible cell for simulation temperatures above 200 K. This is to be expected since the confinement by the framework is much less intense (larger pores). Since the first molecules to be adsorbed have access to the strongest adsorption sites, they however still exhibit a high degree of order resulting in almost “frozen” positioning at lower temperatures. Increasing the simulation temperature counteracts the adsorption energy and leads to a higher mobility of the adsorbate. In the flexible simulations (right panel of Figure 5), however, the confining influence at low loadings is the framework. Raising the temperature therefore does not significantly affect the mobility of the guest. Only at higher loadings, when the MOF is already significantly opened, the mobility of the guest differs between simulation temperatures. At the maximum simulated loading of 22 molecules, neither temperature nor flexibility of the host play a significant role since the packing of CO_2_ is dense and liquid‐like (meaning no pronounced preferential ordering). The experimental maximum loading is difficult to determine exactly. As shown by Bon *et al*.,^[27]^ even after adsorption some parts of the MOF stay closed. By estimating the relative amount of closed MOF one can derive a theoretical maximum loading of 21.5 molecules per formula unit MOF. As this requires *in situ* XRD measurements, this is not commonly done. The maximum loadings of CO_2_ in DUT‐8(Ni) published therefore differ quite significantly.[^17^, ^26^, ^27^, ^30^] From our previous simulations^[34]^ and the experimental results mentioned, we suggest that the maximum loading in experiments will be in the range of 18 to 22 molecules CO_2_ per formula unit MOF (i. e. 27.36 to 33.45 mmol/g). The simulated line widths in this regime are close to 0.1 for temperatures of 300 K and 400 K for the simulations of the rigid variant as well as for the flexible one (see Figure 5). A significant increase in alignment of the CO_2_ molecules is therefore not observed in the flexible framework compared to the rigid one at full loading. The simulations therefore do not support the explanation of the residual chemical shift anisotropy by a framework‐induced force on the adsorbates in the flexible samples. Since the simulation of micrometer sized crystals is well beyond the computationally accessible simulation size regimes, we are bound to the periodic boundary approximation. It is therefore not possible to provide computational proof of the first explanation (fast interparticle exchange). We can only argue against the second explanation via an ordering force applied by the framework. An exemplary comparison of experimental and calculated line shapes is presented in Figure S20 in the Supporting Information. The observed line width of 0.1 (equals. 33.5 ppm), however, perfectly agrees with the measured line widths in the flexible crystals of Sin *et al*.^[30]^ We therefore propose multiple hypotheses: First, the difference in line width in the rigid crystals could be due to differences in the inter‐particle exchange, as already suggested by Sin *et al*.^[30]^ This should be experimentally observable by varying pressure and crystallite sizes (inside the rigid size regime). Secondly, the difference in line width could also be due to different defect concentrations. EPR studies of DUT‐8 have previously shown, that the rigid and flexible crystals also exhibit different defect concentrations.^[20]^ It is imaginable that the higher mobility of guests at defect sites due to reduced confinement is the cause of the decreased line width.

## Conclusions

Through theoretical calculations of ^13^C NMR line widths from MD simulations, we have disproven a possible reason for the difference in residual chemical shift anisotropy of the ^13^C NMR signal of carbon dioxide in rigid and flexible forms of DUT‐8(Ni). The lower anisotropy (narrower signal) of CO_2_ in the rigid DUT‐8(Ni) samples can, according to our simulations, not be explained by an ordering force of the framework on the carbon dioxide molecules. The flexibility of the framework does not influence the orientation of the guest molecules at full loading and can therefore not be the cause of the broader signal (higher degree of order) in the flexible, larger crystallites. The methods and tools used throughout this work are not specific to the investigated MOF and can therefore be used to study confinement of CO_2_ in other materials. By implementing a fast and easy to use algorithm, we enable a wide community to perform direct comparisons between NMR measurements and MD simulation.

## Supporting Information

Supporting Information is available online. All input/output, scripts, structures, plots etc. of the simulations are published as raw data (and visualizations thereof).[^39^, ^40^, ^41^]

The NMR line shape simulation software developed and used throughout this work is available online.^[10]^

## Author Contributions

P.M. ran MD simulations and wrote the line shape simulation software. T.H. contributed to the analysis of the MD simulations by providing ideas for the analysis. P.M. prepared the manuscript and the review process. T.H. acquired the relevant funding and contributed ideas to the manuscript.

## Conflict of interest

The authors declare no conflict of interest.

## Supporting information

As a service to our authors and readers, this journal provides supporting information supplied by the authors. Such materials are peer reviewed and may be re‐organized for online delivery, but are not copy‐edited or typeset. Technical support issues arising from supporting information (other than missing files) should be addressed to the authors.

Supporting Information
