# Supplementary material for: Investigation of CO2 Orientational Dynamics through Simulated NMR Line Shapes
Source: Chemphyschem. 2021 Sep 23;22(22):2336–41. doi: 10.1002/cphc.202100489 (PMC9291905; doi:10.1002/cphc.202100489)
Supplement: Supplementary file 1 — Supporting Information [file CPHC-22-2336-s001.pdf]

ChemPhysChem

Supporting Information

Investigation of CO_2 Orientational Dynamics through Simulated NMR Line Shapes**

Patrick Melix* and Thomas Heine

Author Contributions

P.M. Data curation:Lead; Formal analysis:Lead; Investigation:Lead; Methodology:Lead; Validation:Lead; Visualization:Lead; Writing – original draft:Lead; Writing – review & editing:Lead
T.H. Formal analysis:Supporting; Funding acquisition:Lead; Investigation:Supporting; Methodology:Supporting; Project administration:Lead; Resources:Lead; Supervision:Lead; Validation:Supporting; Writing – original draft:Supporting; Writing – review & editing:Supporting

Contents

Contents 1

1 Methods 2

1.1 Simulation Details 2

1.1.1 Force-Field, LFMM Parameters and Charges 2

1.1.2 MD Setup 2

1.2 NMR Line Shape Calculation 4

1.2.1 Theory 4

1.2.2 Implementation 5

1.2.3 Applicability 6

1.2.4 Accuracy and Sampling 6

2 Results 8

2.1 Simulations 8

2.1.1 Excluded Simulations 8

2.2 Temperature of Subsystems 9

2.4 B Conformer Flexible 11

2.5 Rigid B Conformer 13

2.6 A Conformer 15

3 References 18

1 Methods

1.1 Simulation Details

All input/output, scripts, structures, plots etc. of the simulations are published as raw data (and visualizations thereof).[1]

1.1.1 Force-Field, LFMM Parameters and Charges

Force-Field and LFMM Parameters as well as atomic charges for CO_2 are taken from ref [2].

1.1.2 MD Setup

The extended DL_POLY Classic[3] code presented in ref [4] was used to perform molecular dynamics (MD) simulations. The code was altered to allow execution on multiple processors. A Nose-Hoover thermostat with a relaxation time of 0.1 ps was used. The Velocity Verlet Integration Method was used.

Van-der-Waals potentials were cut off at 11.5 Å. For the empty structures a cutoff of 10.5 Å was used because DL_POLY restricts the cutoff to be smaller than half the perpendicular cell-width, which sometimes occurs during simulations of the empty structures. The error between these two cutoffs was evaluated by comparing obtained energies and was found to be insignificant (less than 1 kcal/mol per formula unit MOF). The Verlet neighbor list shell width was set to 0.5 Å. Electrostatics were calculated using the SPME method with a precision parameter of 10^{-6}. The same cutoff value as for the van der Waals potentials was used for the separation of regions in the Ewald sum.

Using a timestep of 0.5 fs the system was equilibrated during the first 1000 steps while the entire simulation consists of $2*10^6$ steps, resulting in a total simulation time of 1 ns. Every 1000 steps statistics and geometries were printed. A 4x4x4 supercell of conformer B of ref [5] containing a total of 4224 atoms (MOF only) was used for simulations.

For simulations using final states of previous runs the restart mechanism of DL_POLY was used. If the simulation temperature was changed, velocities were scaled using the “restart scale” option. Otherwise, the option “restart noscale” was used. Both use the atomic positions as well as velocities and forces of the final step of the previous simulation to initialize the new simulation. No other information is transferred between the two simulations.

Some simulations needed special setups to maintain system integrity (exploding structures, bond-breaking etc.). All differences are given in Section 1.1.2.4.

1.1.2.1 Flexible Simulations

The N**σ**T ensemble was used by applying a Hoover Barostat using a relaxation time of 1.0 ps with a target pressure of 1 atm.

22 guest Molecules were placed inside a sphere in the open unit cell (alternating linkers, configuration B of ref [5]) using the PackMol[6] code.

Starting structures for lower loadings were generated iteratively using the following procedure:

1. N**σ**T simulation at 1 K for 2000 steps (including 100 equilibration steps) with a barostat relaxation time of 5.0 ps.
2. Final configuration is stored as starting structure for the long simulation run.
3. Removal of every 64th guest molecule, resulting in one guest molecule removed from every MOF-void. If no guest molecules remain, exit.
4. Insert new structure with lower loading into 1.

1.1.2.2 Rigid Simulations

The NVT ensemble was used. Starting configurations and cells were derived from the flexible simulation at a loading of 22 Molecules. The final structure and cell of the fully loaded, flexible simulation was copied. Then every 64th guest molecule, corresponding to one molecule per MOF void, was removed iteratively to generate all lower loadings. This proceeding leads to an almost homogeneous distribution of guest molecules in the starting configurations. The positions of MOF atoms were not altered in this process.

1.1.2.3 Conformer A Simulations

One to 21 guest Molecules were placed inside a sphere in the open unit cell (all linkers up or down, configuration A(op) of ref [5]) using the PackMol[6] code. Otherwise, the same setup as for the "Flexible" simulations was used. Technically a 2x4x4 supercell was used (with a rotated cell to align the structure in a similar fashion as in the conformer **B** simulations), but the number of atoms is the same as for the other simulations since the unit cell of conformer A is twice the size of conformer **B**.

1.1.2.4 Differing MD Setups

Rigid NVT simulations were performed as described above.

Flexible **B** conformer simulations:

- 200K:
  - 2, 8 and 9 Molecules: Preceding NVT simulation.
  - 3 and 5 Molecules: Restarted from ref [2] 200K.

- 300K:
  - 2 and 8 Molecules: Restarted from ref [2] 300K.
  - 4 Molecules: Preceding NVT simulation. Using 1.0 ps and 5.0 ps for thermostat and barostat.
  - 7 Molecules: Preceding 100K and 200K simulations with 200,000 steps.
- 400K:
  - 2 Molecules: Restarted from 300K.
  - 4 Molecules: Restarted from ref [2] 300K.
  - 7 Molecules: Restarted from ref [2] 200K using 10.000 equilibration steps.
  - 8 Molecules: Restarted from ref [2] 300K. Intermediate 350K simulation.

A conformer simulations:

- Loadings of 22 molecules could not be stabilized at any temperature.
- 300K:
  - 20 and 21 Molecules: Restarted from 200K.
  - 3 Molecules: Restarted from 400K.
  - 8 Molecules: Restarted from 200K with intermediate 250K simulation.
- 400K:
  - 6-8, 11-19 Molecules: Restarted from 300K.
  - 20 and 21 Molecules: Restarted from 200K.

The differing MD setups were introduced in an ad hoc way. Based on our previous works, ample experience with bad initial guesses was already accumulated. We therefore used the trial-error based results of how to achieve usable initial guesses also in this work. However, the tricks used (preceding NVT, restarting from lower temperatures, more equilibration steps) are frequently used methods to improve the starting configuration in MD simulations. We do not sample different starting configurations, so an influence of these cannot be excluded, but is very unlikely for the conclusions drawn based on the convergence data of the individual calculations.

The differing thermostat and barostat relaxation times are easily explained by the reuse of an older input file as a template for the intermediate steps and are therefore not based on any argument. Since only atomic positions, forces and velocities are taken over from these very short preliminary simulations as an initial guess, this difference is irrelevant to the results of the following simulation. The number of simulation steps in the production simulation was furthermore the same for all systems.

The breaking of structures is purely an artifact of bad initial guesses of the atomic positions and is frequently encountered in all kinds of MD simulations. The techniques mentioned above are some of the tricks used to help generate more reasonable starting structures.

1.2 NMR Line Shape Calculation

1.2.1 Theory

The theory for the NMR line shape calculations of linear molecules was first proposed by Eisbein *et al.* in ref [7]. The underlying theory is published in E. Eisbein's PhD-Thesis[8] (in German). We reproduce parts of the theory here to make it accessible to the interested reader.

For an anisotropic, linear molecule like CO_2, the shielding tensor **σ** contains two contributions. One for parallel ($\delta_{||}$) and one for perpendicular ($\delta_{\perp}$) orientation to the external magnetic field $\overrightarrow{B_0}$:

$$\boldsymbol{\sigma} = \begin{pmatrix} \delta_{||} & 0 & 0 \\ 0 & \delta_{\perp} & 0 \\ 0 & 0 & \delta_{\perp} \end{pmatrix} \tag{1}$$

Since the chemical shifts δ of CO_2 for parallel and perpendicular orientation are known ($\delta_{||} = -90$ ppm and $\delta_{\perp} = 245$ ppm, using TMS as reference[9]) and the chemical surrounding is anisotropic in only one spatial dimension, the chemical shift can be expressed as

$$\delta = \delta_{\perp} - \left(\delta_{\perp} - \delta_{||}\right)\left[\frac{\overrightarrow{B_0} \cdot (\vec{r} \otimes \vec{r}) \cdot \overrightarrow{B_0}}{\overrightarrow{B_0} \cdot \overrightarrow{B_0}\, \vec{r} \cdot \vec{r}}\right] \tag{2}$$

with $\vec{r}$ being the orientation vector of the molecule. By setting $\delta_{\perp} = 1$ and $\left(\delta_{\perp} - \delta_{||}\right) = \Delta = 1$ (therefore $\delta_{||}$ is set to be zero) the chemical shift can be expressed as a function of the orientation vector $\vec{r}$ with respect to the magnetic field $\overrightarrow{B_0}$. This can be done since both $\delta_{\perp}$ and $\delta_{||}$ are experimental constants that are measured with TMS as a reference. Normalized here is therefore the difference between the two, which is Δ. This is however just a mathematical trick to remove constants and has no further effect than to make all results relative to the distance of $\delta_{\perp}$ and $\delta_{||}$.

During an NMR experiment, the vector $\vec{r}$ is not static. The measured signal therefore represents a mean chemical shift $\bar{\delta}$ that can be expressed as

$$\bar{\delta} = 1 - \frac{\overrightarrow{B_0}}{\overrightarrow{B_0} \cdot \overrightarrow{B_0}} \cdot \left[\frac{1}{N}\sum_{i=1}^{N} \frac{\vec{r_i} \otimes \vec{r_i}}{\vec{r_i} \cdot \vec{r_i}}\right] \cdot \overrightarrow{B_0} \tag{3}$$

The bracket in equation (3) is a second order tensor that can be represented by a 3x3 matrix. Diagonalizing this matrix and feeding it to the powder pattern computation method of Alderman *et al.*,[10] results in an efficient way to calculate NMR line shapes.

For two cases, the resulting line shape can be derived analytically: Fully aligned molecules (perfect crystal) and a homogeneous gas phase (perfectly randomized orientations). For the former, the tensor in equation (3) has two eigenvalues equal to zero and one equal to one. The line shape can then be expressed as

$$I(\overline{\delta}) = \frac{1}{4\sqrt{1-\overline{\delta}}}, \quad (4)$$

with $I(\overline{\delta})$ being the intensity. For the gas phase, the eigenvalues of the tensor are all $1/3$, resulting in a single peak of infinite height at position $2/3$.

1.2.2 Implementation

We implemented the calculation of NMR powder pattern line shapes in a Python module. Our implementation differs significantly from the one presented by Eisbein *et al*.[7, 8] By using the NumPy library[11] we were able to reduce the computational cost significantly and can provide a more general and easier to use implementation. By feeding a list of orientation vectors to the software, the NMR powder pattern line shapes are now easily obtainable for any system. We published the source code under a MIT license on GitHub and provide a citable reference using Zenodo.[12]

All line shapes calculated by our code are normalized so that the integral over the signal equals one.

As a validation of our implementation, we provide the simulation of aligned vectors (perfect crystal) and random vectors (gas phase) in Figure S1. The inputs consisted of 10 times the same vector ("frozen") and 10^6 randomly generated vectors (generated using the NumPy function `*numpy.random.normal(size=3)*`, "gaseous").

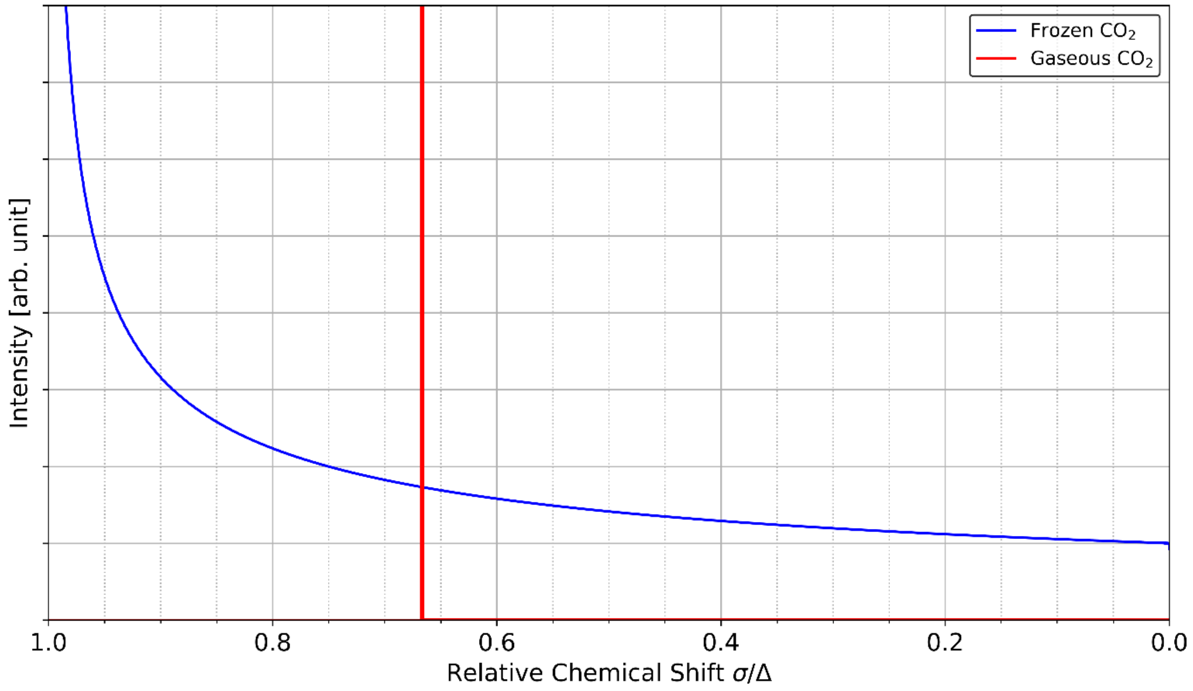

Figure S1: Calculated line shapes for frozen (blue) and gaseous (red) arrangements of molecules. Calculated using 10.000 bins and 256 intersections in the Alderman interpolation scheme. Reproduced under the terms of the Creative Commons Attribution 4.0 License.[1]

1.2.3 Applicability

This approach is only valid when the host has a neglectable influence on the guests shielding tensor. In the case of CO_2 in DUT-8(Ni) this is expected to be the case. We verified our expectation by calculating shielding tensors for free CO_2 and CO_2 near a $Zn_2(OOCH)_4$ cluster, adjacent to one of the

undercoordinated Zn atoms. We used the ADF2019 Software,[13] employing the PBE functional with a TZ2P-J basis set, Grimme's D3(BJ) dispersion correction and relativistic spin-orbit effects with the ZORA formalism. Since only the effect of the neighboring metal center on the shielding tensor is relevant here, the accuracy of the method itself is not relevant. Using this approach, we found a neglectable influence on the shielding tensor of the CO_2 carbon atom. The principal components of the shielding tensor changed from (-69.5 ppm; -69.5 ppm; 286.4 ppm) for the free CO_2 molecule to (-67.5 ppm; -63.1 ppm; 280.0 ppm) for the coordinated molecule.

The linearity of the CO_2 molecules is almost exactly maintained during our MD simulation, the observed O-C-O angles exhibit no significant deviation from 180 degree.

1.2.4 Accuracy and Sampling

In our calculations we used a number of $N = 256$ intersections of the triangular interpolation grid.[10]

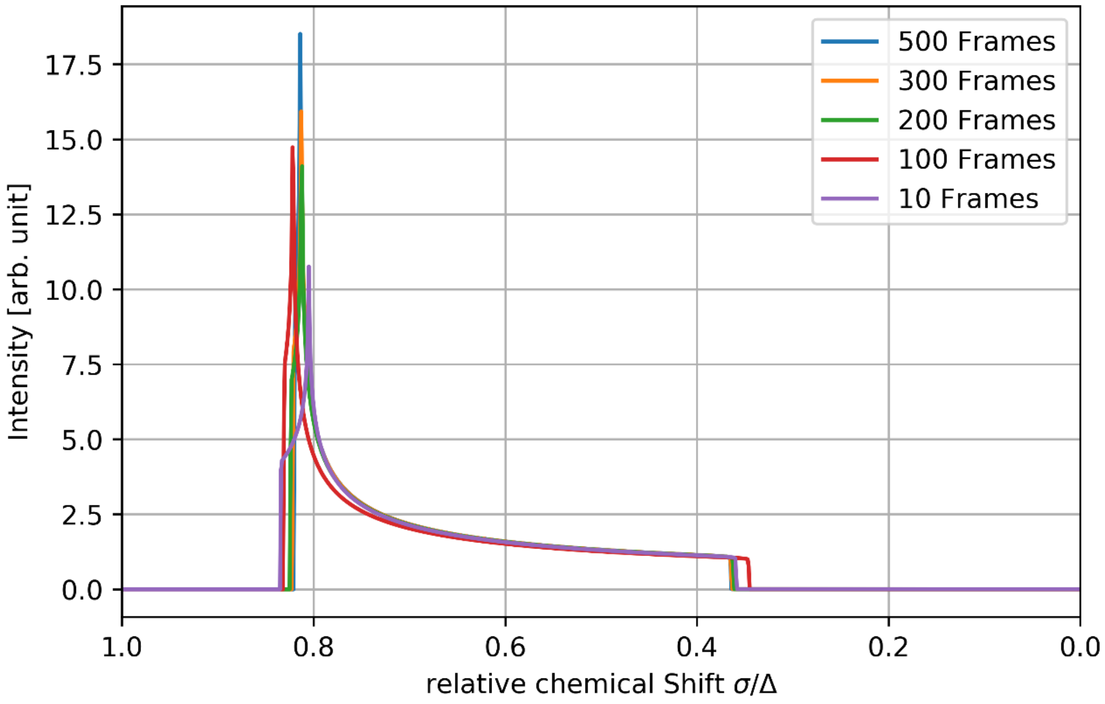

Figure S2:Calculated line shapes from the rigid 1 Molecule simulation at 300K. The number of simulation frames analyzed is given in the legend for each line. Reproduced under the terms of the Creative Commons Attribution 4.0 License.[1]

The calculated line width is influenced by computational parameters such as simulation time and initial CO_2 locations. For that reason, we sampled the lowest loading at 300K for convergence of the calculated line shape (Figure S2). Based on these results, all Oxygen-Oxygen vectors of the last 300 frames of our MD simulations were sampled. As can be seen, the main peak position remains

unaltered when more than 200 frames are considered. The entire line shape is converged with 300 frames being analyzed. With increasing loading, the sampling of CO_2 positions automatically increases, as more CO_2 are present in the simulation. Therefore, all higher loadings will converge the calculated line shape earlier.

Furthermore, we make sure that the temperatures, cells and pressures of all MD simulations are well converged in the analyzed timeframe. We also calculate the instantaneous temperatures of the MOF and CO_2 based on the velocities to confirm this (Section 2.2). What we do not do, is to sample multiple starting configurations. For low loadings and temperatures, we do believe that this is the reason for the observed discontinuities of the line width (see Figure 5). Therefore, we also do not interpret these results or draw conclusions from single data points. We acknowledge the lack of sampling in this low loading, low temperature regime, argue however that it is not relevant for the conclusions drawn.

To ensure that the simulation setup is able to reproduce a gas-like NMR signal and does not artificially exclude narrow line widths, as observed in experiments for the rigid crystals, a pure CO_2 trajectory was sampled. We therefore ran one MD simulation using the final positions of CO_2 molecules and the simulation cell of the **B** rigid, 400 K and 10 guest molecules simulation. The same simulation setup as for the simulation including the framework and the exact same sampling methods were applied.

The resulting line width is 0.004, the simulated line shape is given in Figure S3. This clearly proves that the model can predict a gas-like line width using my simulation setup.

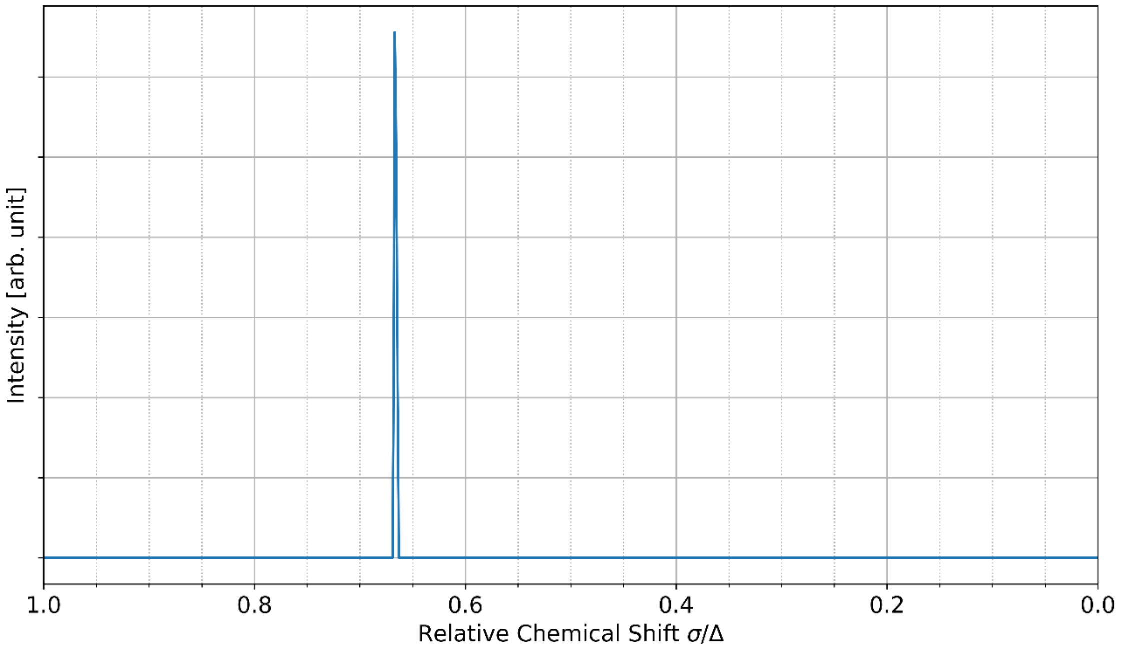

Figure S3: Calculated line shape of the rigid 10 Molecule simulation at 400K without the framework. Reproduced under the terms of the Creative Commons Attribution 4.0 License.[1]

2 Results

We collect here the results not presented in the main text.

2.1 Simulations

2.1.1 Excluded Simulations

We excluded one simulation from analysis: The **A** conformer simulation with a loading of 8 molecules at 400K. It was restarted from the 300K simulations, which already showed some linker detachment. System could not be stabilized resulting in total structural decomposition.

2.2 Temperature of Subsystems

To assert the validity of the observed line widths, we calculated the instantaneous temperature of the MOF, the guest molecules and the entire system based on the final atomic velocities. The same methodology as in our previous work was used.[14]

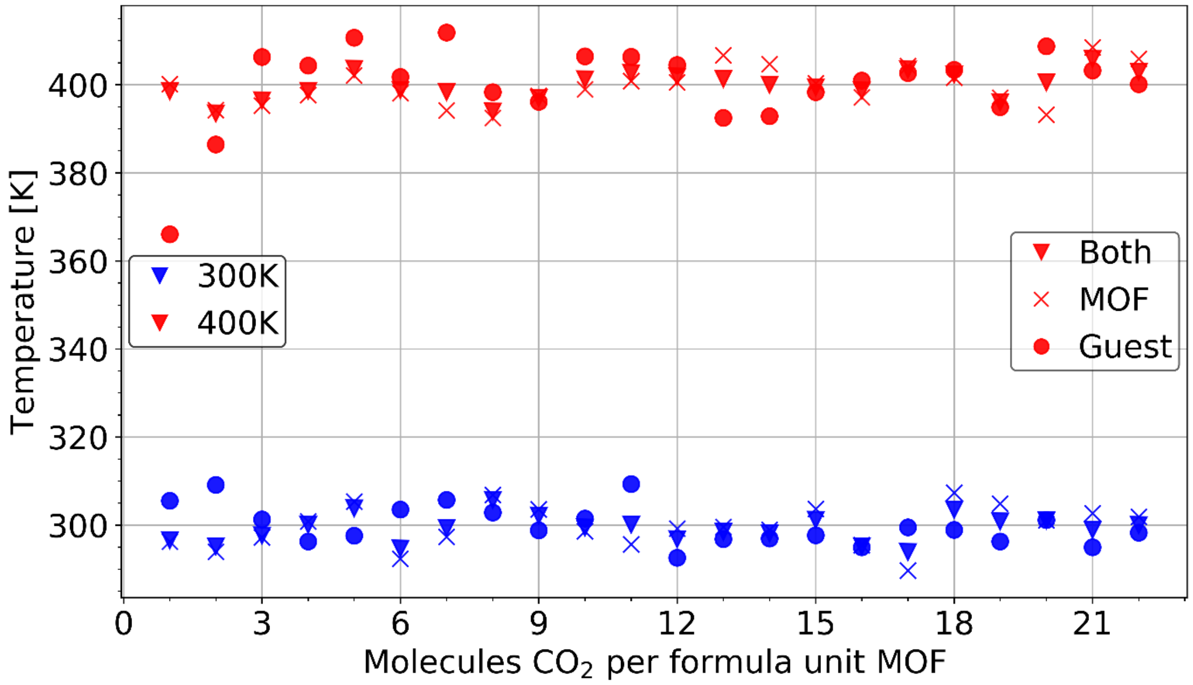

Figure S4: Instantaneous temperature calculated from atom velocities for the entire system (triangles), the MOF (stars) and the guest only (crosses) for the 300 K (orange) and 400 K (red) flexible ***B*** *conformer simulations. Reproduced under the terms of the Creative Commons Attribution 4.0 License.[1]*

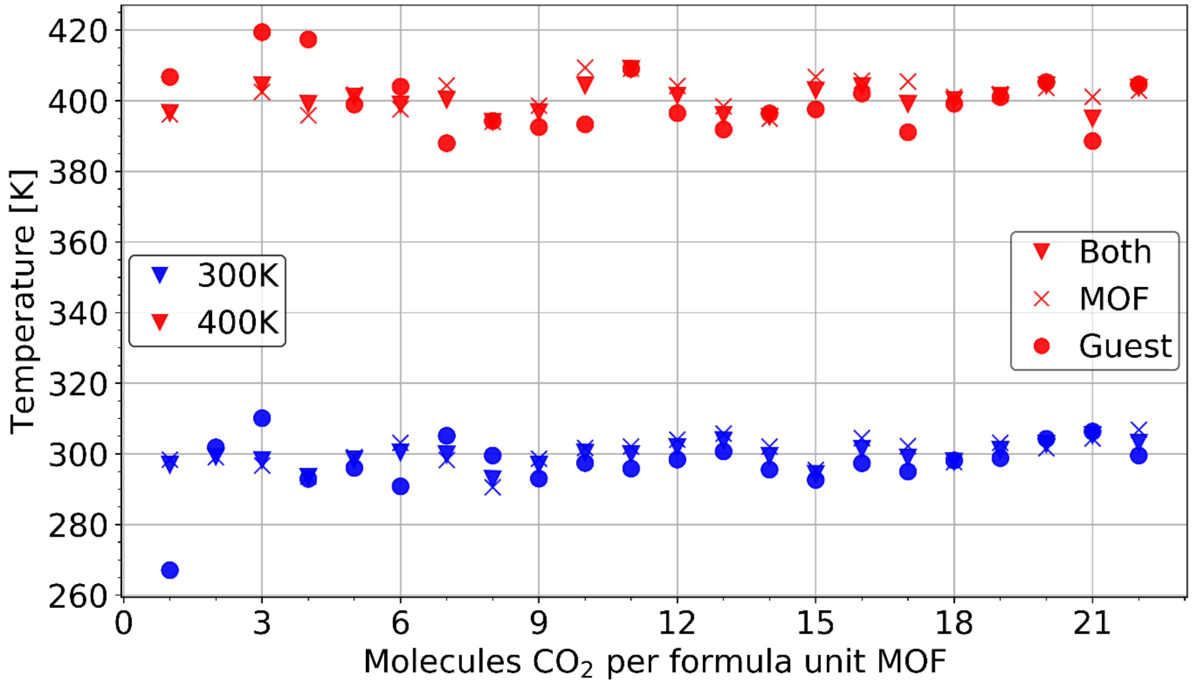

Figure S5: Instantaneous temperature calculated from atom velocities for the entire system (triangles), the MOF (stars) and the guest only (crosses) for the 300 K (orange) and 400 K (red) rigid ***B*** *conformer simulations. Reproduced under the terms of the Creative Commons Attribution 4.0 License.*[1]

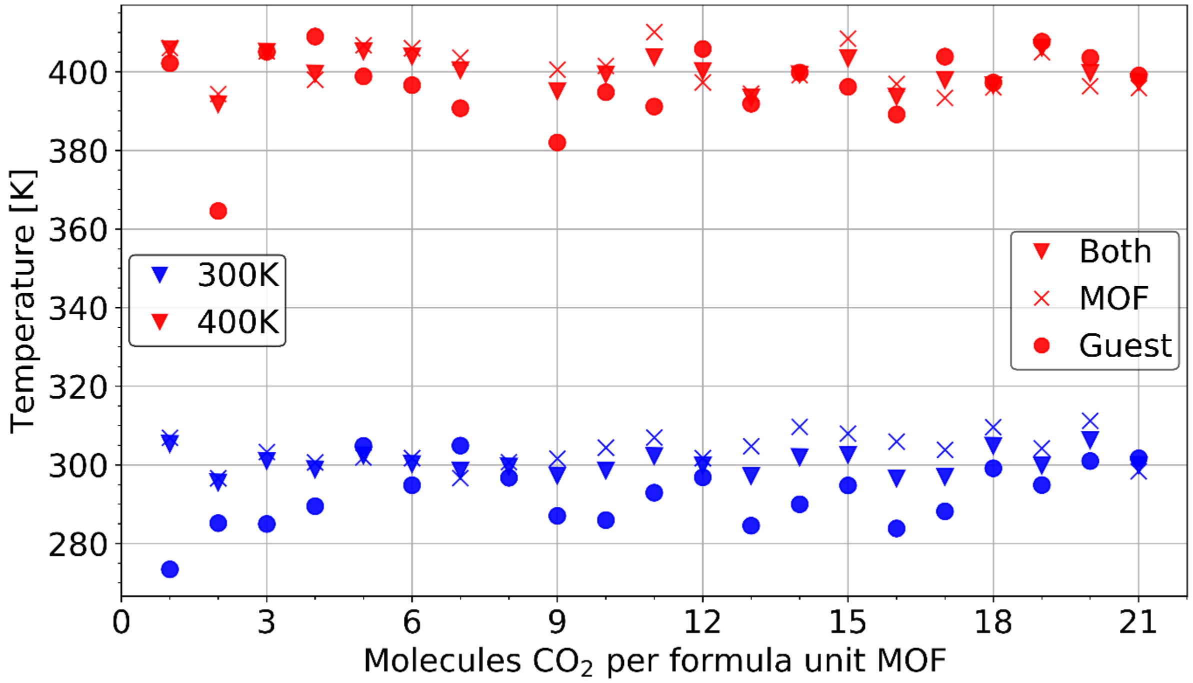

Figure S6: Instantaneous temperature calculated from atom velocities for the entire system (triangles), the MOF (stars) and the guest only (crosses) for the 300 K (orange) and 400 K (red) ***A*** *conformer simulations. Reproduced under the terms of the Creative Commons Attribution 4.0 License.*[1]

2.4 B Conformer Flexible

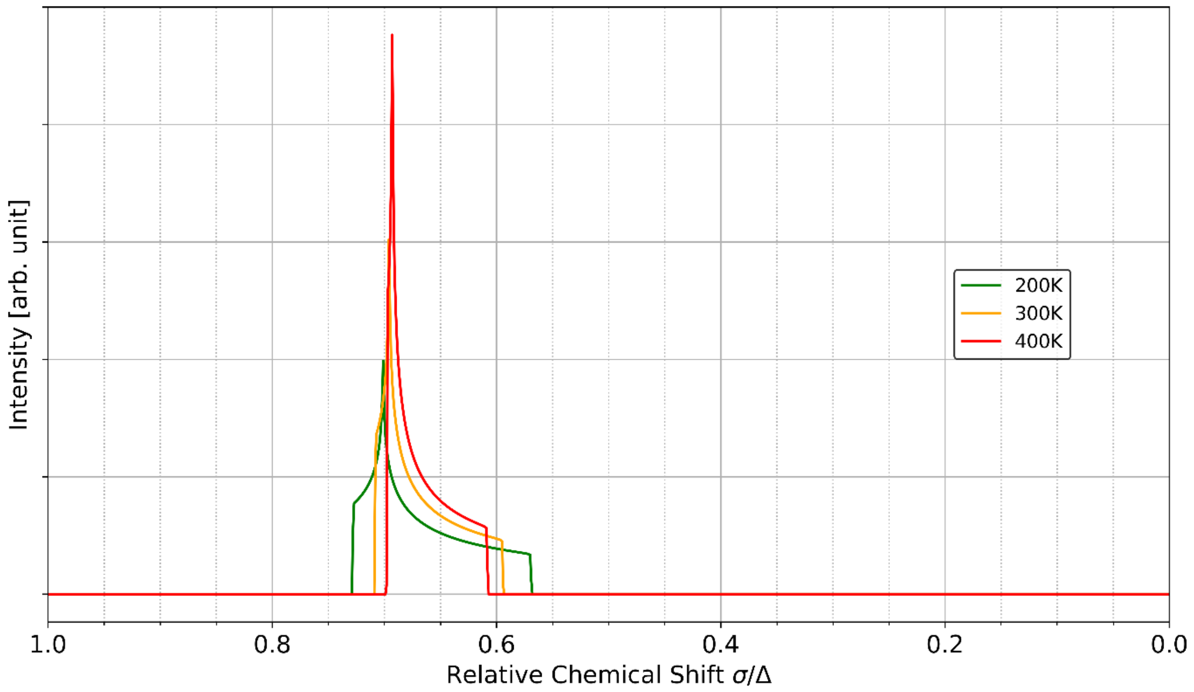

*Figure S7: Calculated line shapes of 20 Molecules CO_2 per formula unit MOF at 200K (green), 300K (orange) and 400K (red) in the flexible **B** conformer simulations. Reproduced under the terms of the Creative Commons Attribution 4.0 License.[1]*

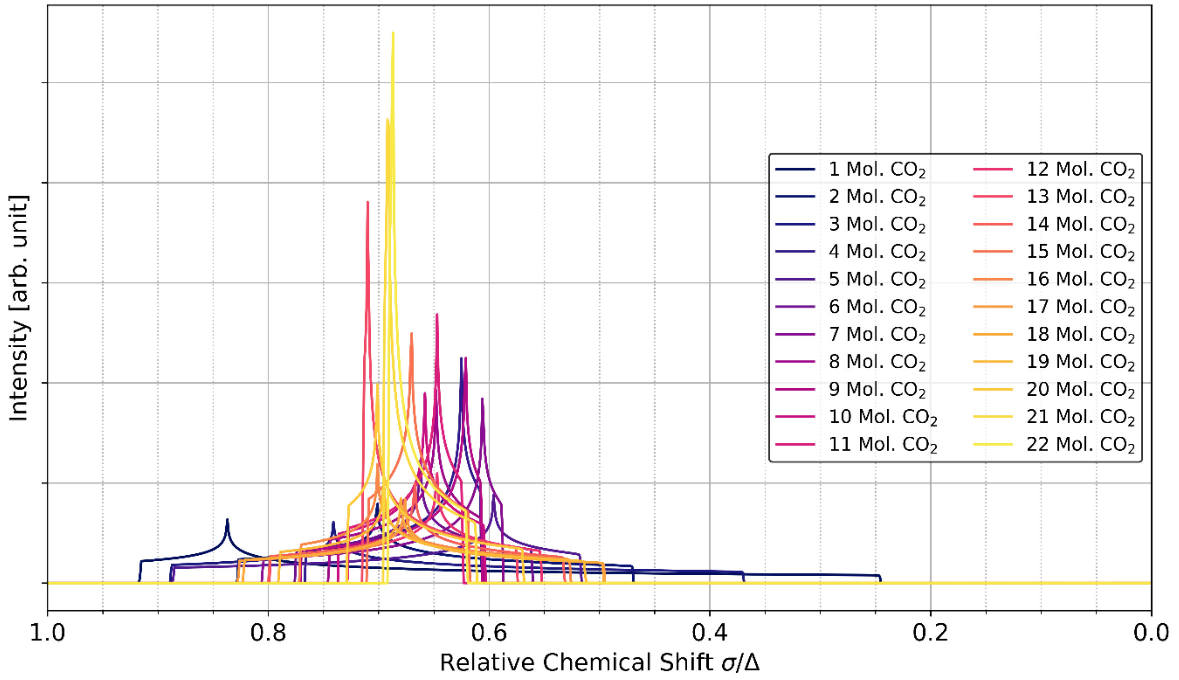

*Figure S8: Calculated line shapes of CO_2 for all loadings at 200K in the flexible **B** conformer simulations. Reproduced under the terms of the Creative Commons Attribution 4.0 License.[1]*

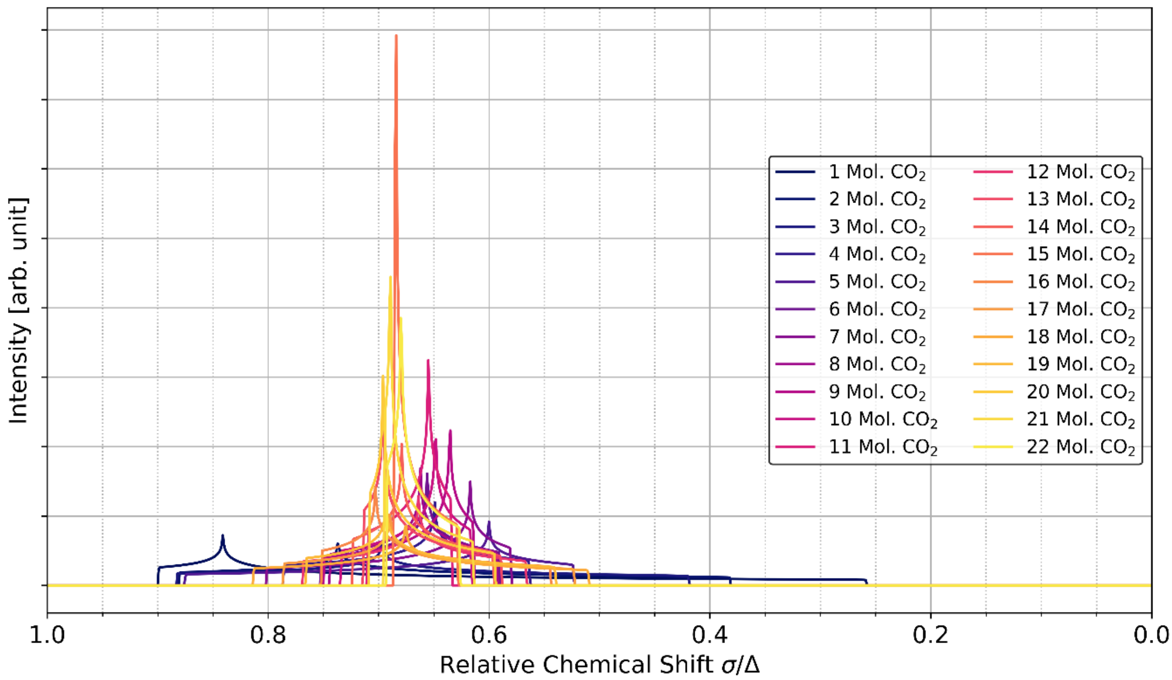

Figure S9: Calculated line shapes of CO_2 for all loadings at 300K in the flexible ***B*** *conformer simulations. Reproduced under the terms of the Creative Commons Attribution 4.0 License.*[1]

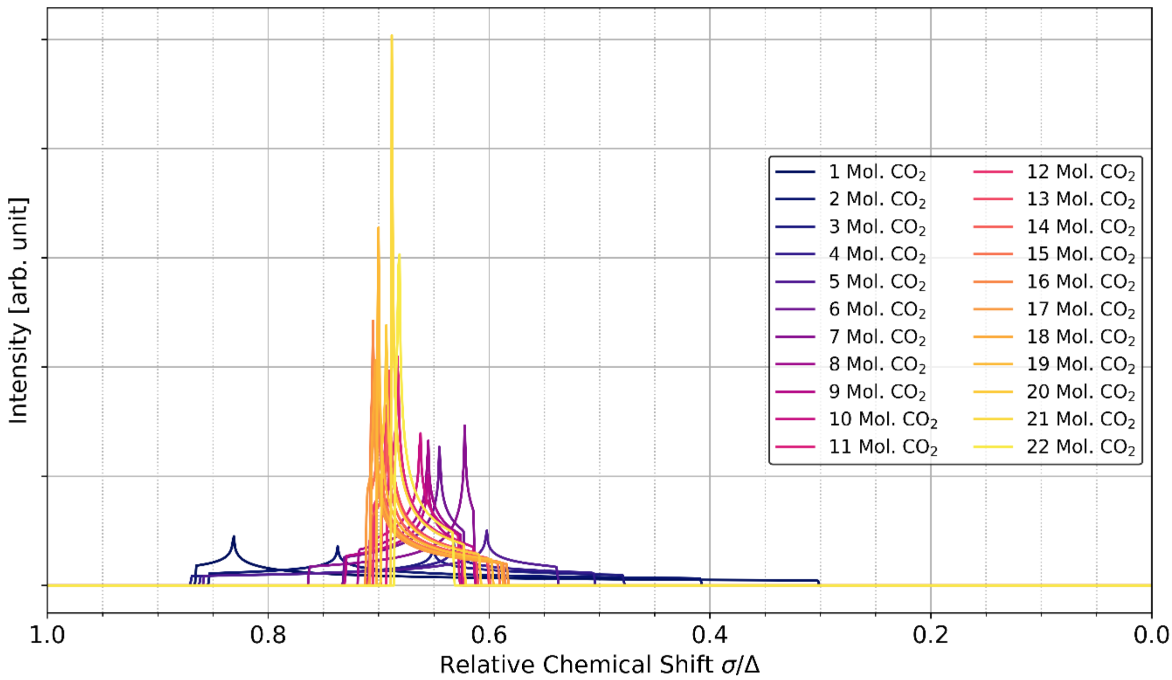

Figure S10: Calculated line shapes of CO_2 for all loadings at 400K in the flexible ***B*** *conformer simulations. Reproduced under the terms of the Creative Commons Attribution 4.0 License.*[1]

2.5 Rigid B Conformer

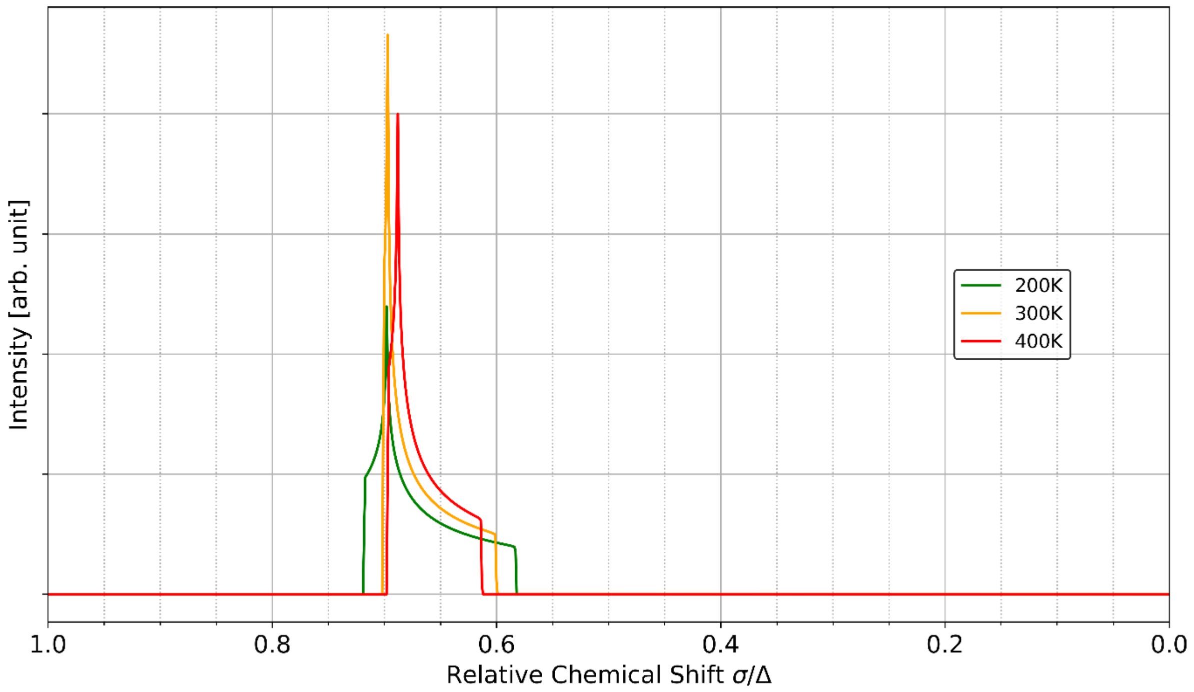

*Figure S11: Calculated line shapes of 20 Molecules CO_2 per formula unit MOF at 200K (green), 300K (orange) and 400K (red) in the rigid **B** conformer simulations. Reproduced under the terms of the Creative Commons Attribution 4.0 License.*[1]

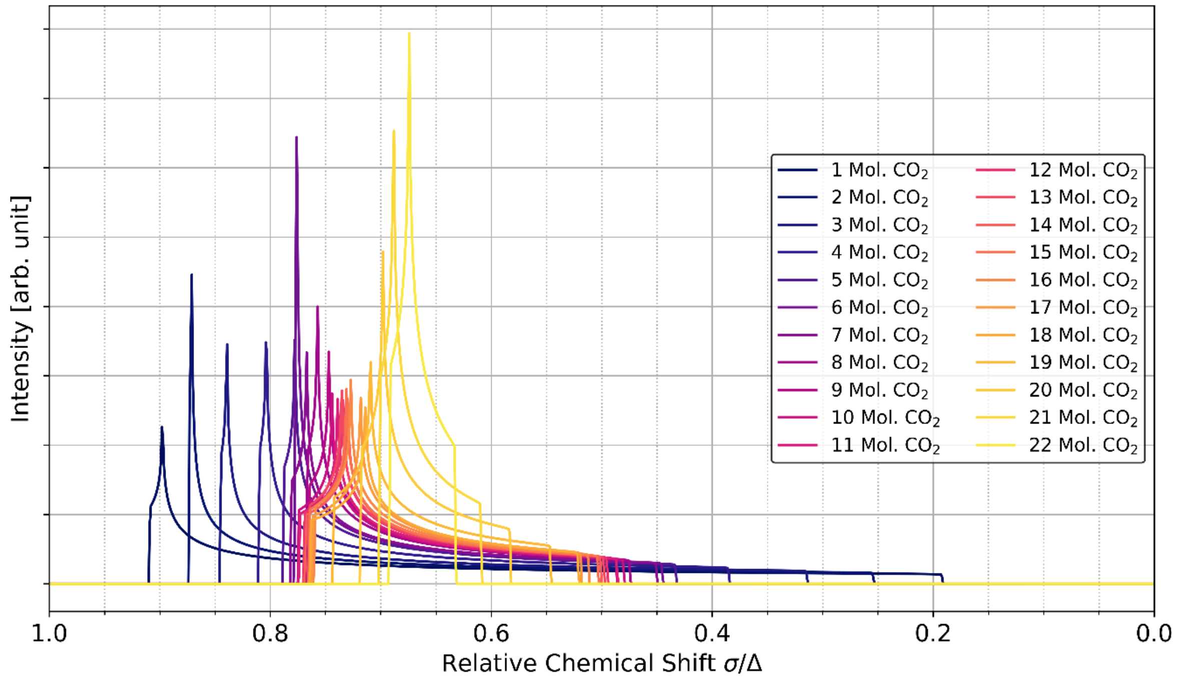

*Figure S12: Calculated line shapes of CO_2 for all loadings at 200K in the rigid **B** conformer simulations. Reproduced under the terms of the Creative Commons Attribution 4.0 License.*[1]

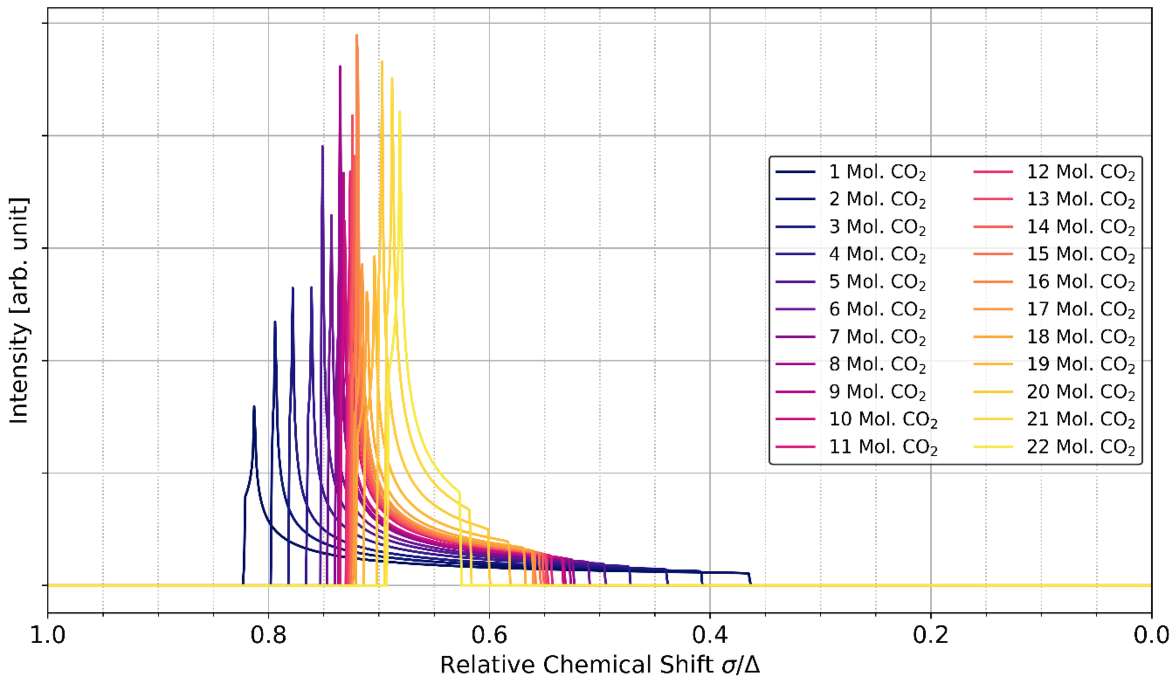

Figure S13: Calculated line shapes of CO_2 for all loadings at 300K in the rigid ***B*** *conformer simulations. Reproduced under the terms of the Creative Commons Attribution 4.0 License.*[1]

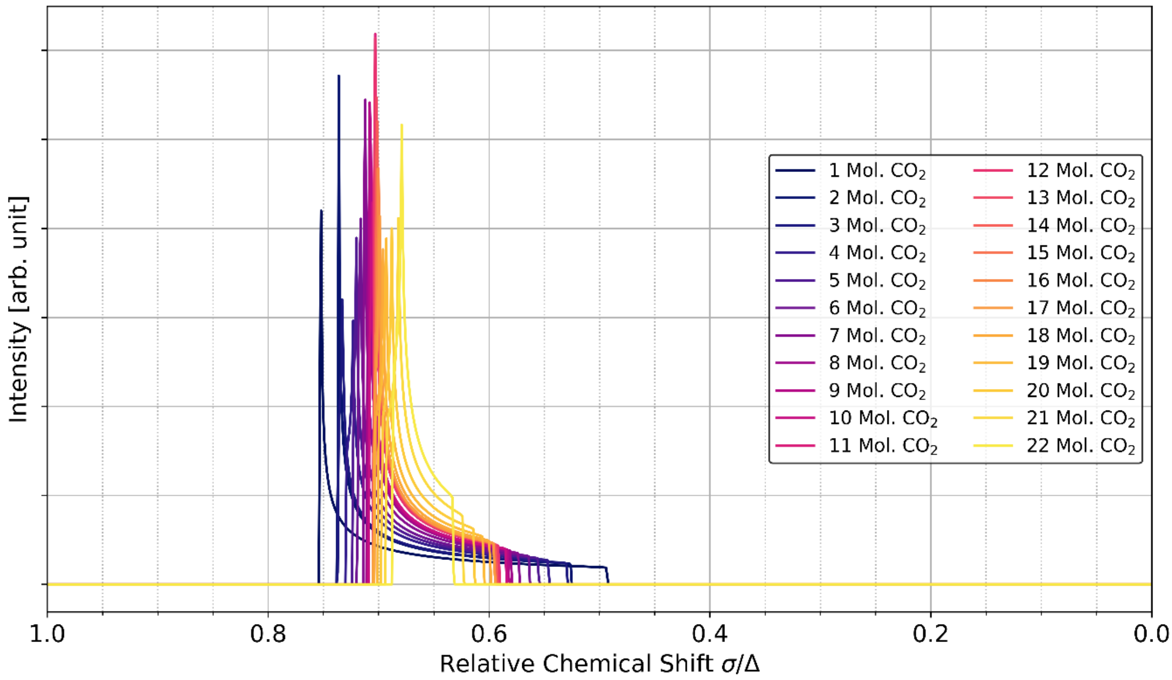

Figure S14: Calculated line shapes of CO_2 for all loadings at 400K in the rigid ***B*** *conformer simulations. Reproduced under the terms of the Creative Commons Attribution 4.0 License.*[1]

2.6 A Conformer

The simulated line widths of the rigid and flexible **B** conformer simulations are given in the main text. In Figure S15 the relative line widths of the **A** conformer simulations and the respective cell volume are given. Intermediate loadings are not experimentally observable since the adsorption occurs in a stepwise fashion. The observed closing of the structure in our MD simulation therefore does not contradict the experimental observation of rigid crystals, since the simulations employ a ensemble using a constant number of particles. As observed in ref [2], the host-guest interactions are larger than the energy penalty for closing the MOF and therefore enable the closing of the structure even in the **A** conformer.

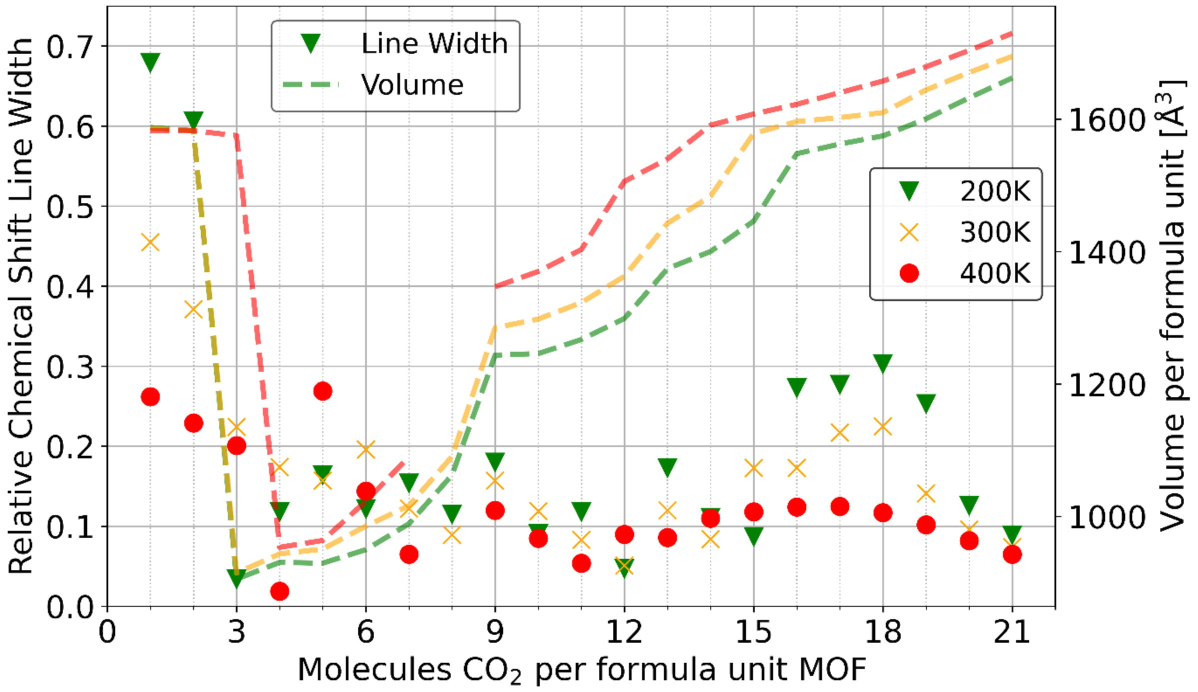

*Figure S15: Relative chemical shift line widths with respect to the number of CO_2 molecules loaded per formula unit MOF at 200K (green), 300K (yellow) and 400K (red) simulation temperature in the **A** conformer simulations. Cell volumes given as dashed lines. Reproduced under the terms of the Creative Commons Attribution 4.0 License.[1]*

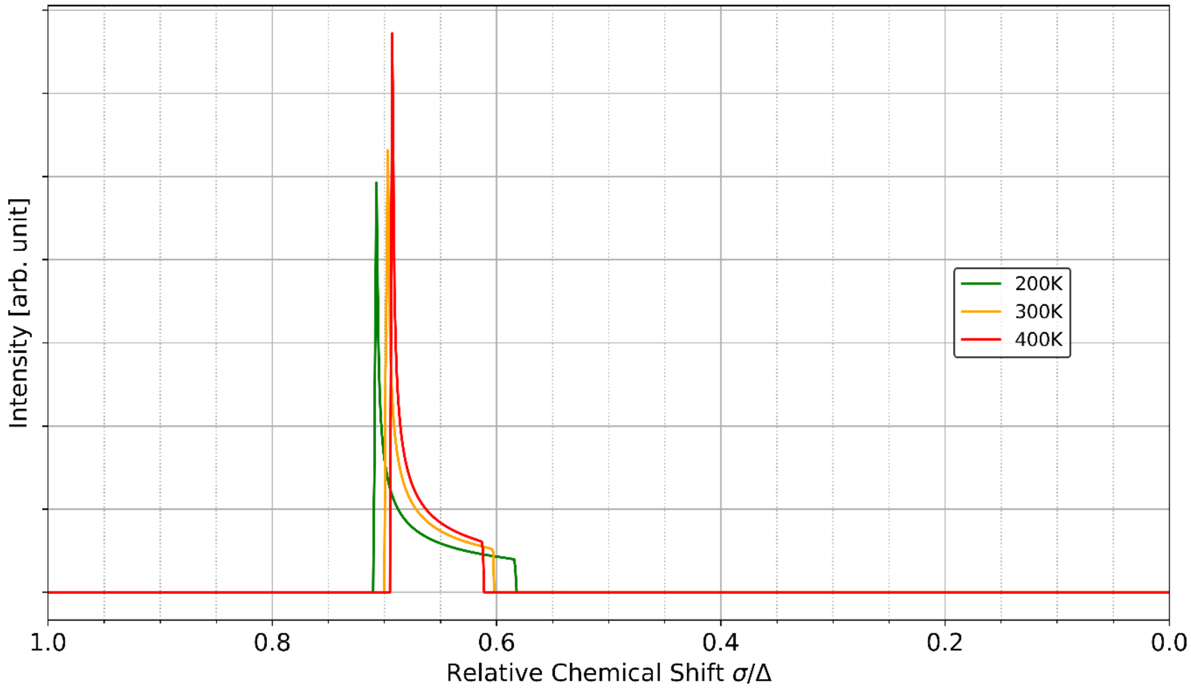

*Figure S16: Calculated line shapes of 20 Molecules CO_2 per formula unit MOF at 200K (green), 300K (orange) and 400K (red) in the **A** conformer simulations. Reproduced under the terms of the Creative Commons Attribution 4.0 License.[1]*

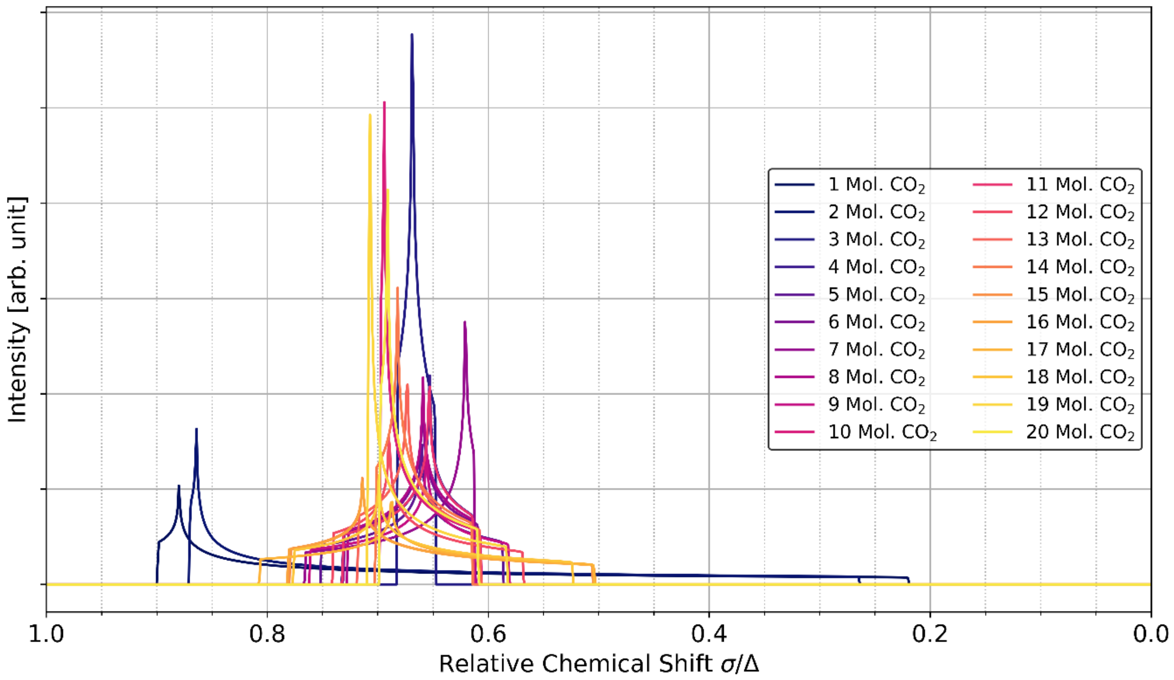

*Figure S17: Calculated line shapes of CO_2 for all loadings at 200K in the **A** conformer simulations. Reproduced under the terms of the Creative Commons Attribution 4.0 License.[1]*

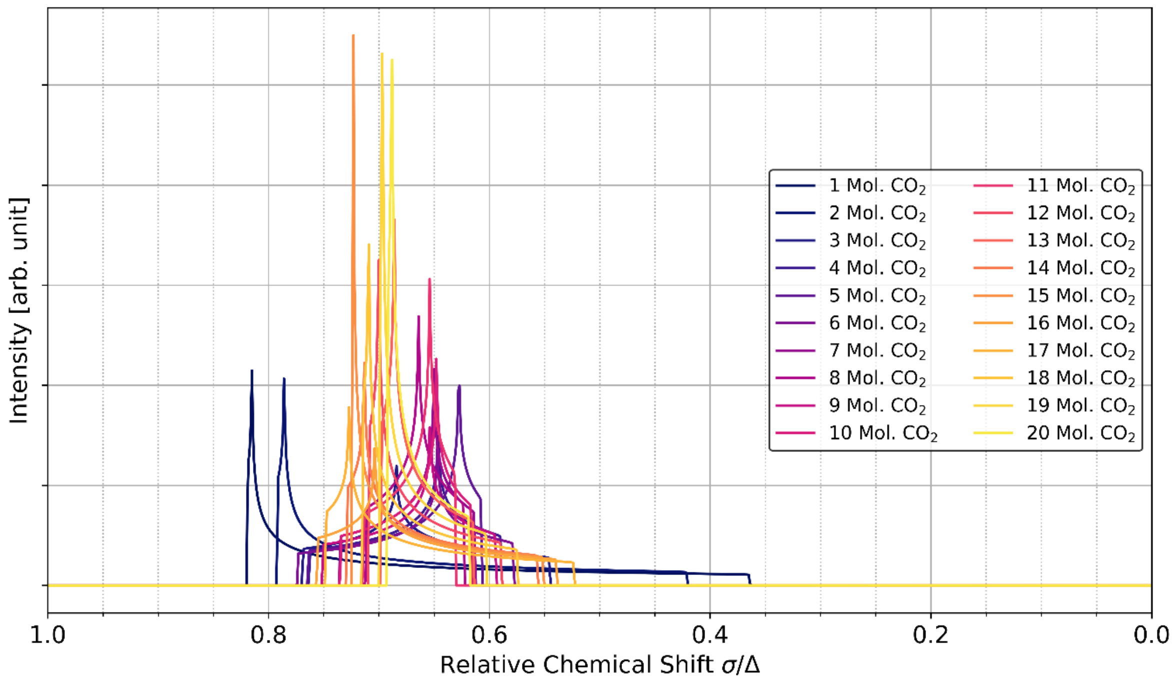

*Figure S18: Calculated line shapes of CO_2 for all loadings at 300K in the **A** conformer simulations. Reproduced under the terms of the Creative Commons Attribution 4.0 License.[1]*

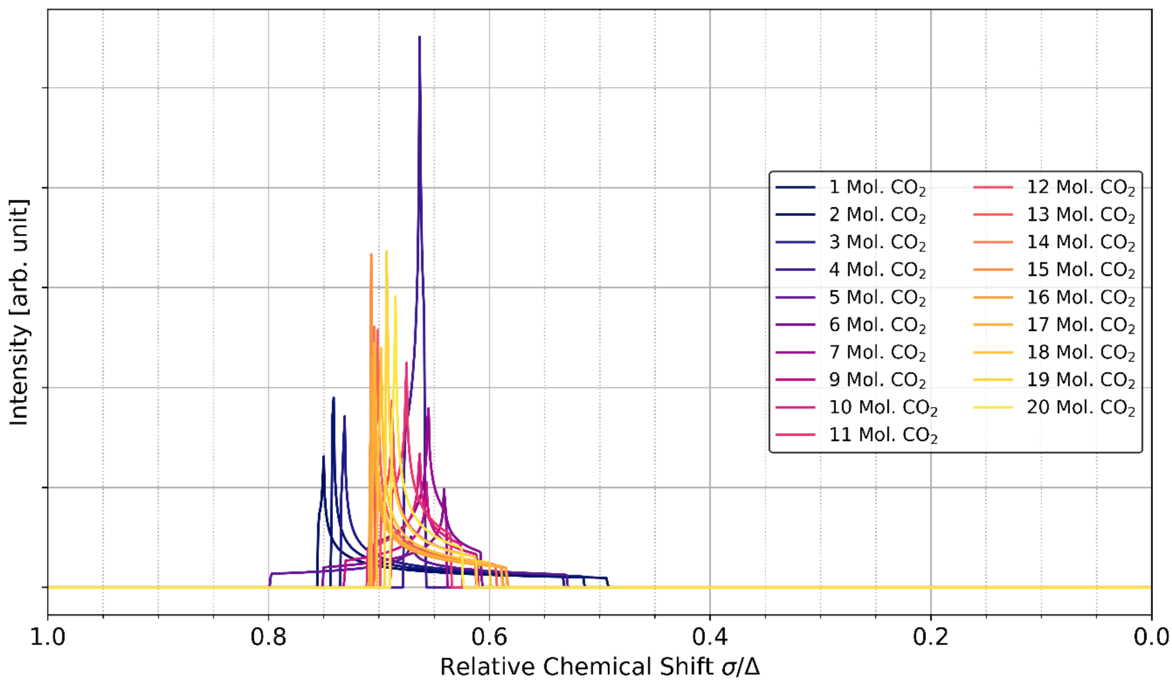

*Figure S19: Calculated line shapes of CO_2 for all loadings at 400K in the **A** conformer simulations. Reproduced under the terms of the Creative Commons Attribution 4.0 License.[1]*

2.7 Visual Comparison Experimental and Calculated Line Shapes

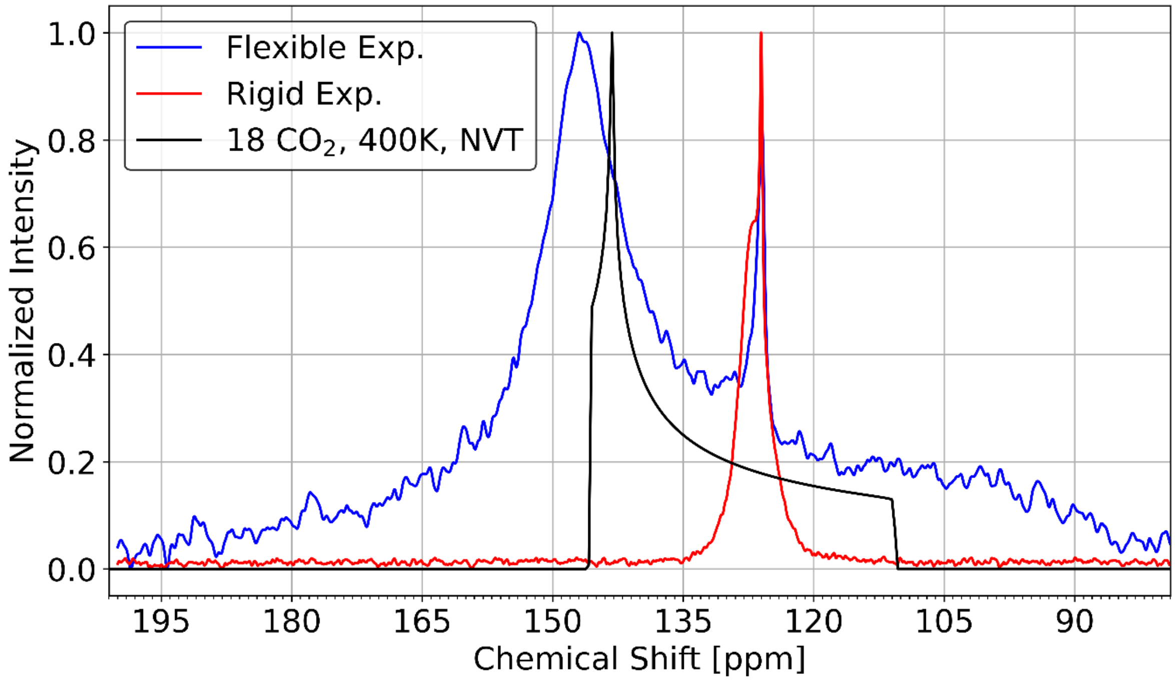

*Figure S20: Measured ^{13}C NMR spectra of CO_2 adsorbed in flexible (blue) and rigid (red) samples of DUT-8(Ni) at 215 K. Flexible sample measured at a partial CO_2 pressure of 5.1bar (2:1 CO_2/CH_4 mixture, no CH_4 adsorption) and rigid sample measured at a CO_2 pressure of 6.6bar. Both samples are in the **op** state at these experimental conditions. Calculated line shape of rigid cell 400K simulation with 18 molecules of CO_2 (black). Adapted with permission from Sin et al., Langmuir, 2019, 35 (8), 3162-3170. Copyright 2019 American Chemical Society.[15]*

In Figure S20 the experimental NMR signals for rigid and flexible crystals from Sin et al.[15] are plotted alongside the calculated line shape of the rigid conformer **B**, 400 K, 18 molecule simulation. Note that the experimental peak of gaseous CO_2 is shifted by approximately 7 ppm to lower shifts compared to the ideal position of 133.3 ppm, this experimental difference is not corrected here.

3 References

[1] P. Melix **2021** Investigation of CO_2 Orientational Dynamics through Simulated NMR Line Shapes, Zenodo, DOI 10.5281/zenodo.4905822.

[2] P. Melix, F. Paesani, T. Heine, *Adv. Theory Simul.* **2019**, *2*, 1900098.

[3] I.T. Todorov, W. Smith, K. Trachenko, M.T. Dove, *J. Mater. Chem.* **2006**, *16*, 1911 -1918.

[4] J. Cirera, V. Babin, F. Paesani, *Inorg. Chem.* **2014**, *53*, 11020-11028.

[5] P. St. Petkov, V. Bon, C.L. Hobday, A.B. Kuc, P. Melix, S. Kaskel, T. Düren, T. Heine, *Phys. Chem. Chem. Phys.* **2019**, *21*, 674-680.

[6] J.M. Martinez, *J. Comput. Chem.* **2009**, *30*, 2157-2164.

[7] V. Bon, J. Pallmann, E. Eisbein, H.C. Hoffmann, I. Senkovska, I. Schwedler, A. Schneemann, S. Henke, D. Wallacher, R.A. Fischer, et al., *Microporous Mesoporous Mater.* **2015**, *216*, 64-74.

[8] E. Eisbein Molekulardynamische Simulationen zu Wirt-Gastsystemen auf der Basis von Metallorganischen Gerüstverbindungen. TU Dresden. Phdthesis.

[9] A.J. Beeler, A.M. Orendt, D.M. Grant, P.W. Cutts, J. Michl, K.W. Zilm, J.W. Downing, J.C. Facelli, M.S. Schindler, W. Kutzelnigg, *J. Am. Chem. Soc.* **1984**, *106*, 7672 -7676.

[10] D. W. Alderman, Mark S. Solum, David M. Grant, *J. Chem. Phys.* **1986**, *84*, 3717-3725.

[11] T. Oliphant **2006-** NumPy: A guide to NumPy. http://www.numpy.org/.

[12] P. Melix **2019** *NMRLineshape*, Zenodo, DOI 10.5281/zenodo.3450684.

[13] SCM ADF, Amsterdam, The Netherlands. http://www.scm.com.

[14] P. Melix and T. Heine, *J. Phys. Chem. C* **2020**, *124*, 11985-11989.

[15] M. Sin, N. Kavoosi, M. Rauche, J. Pallmann, S. Paasch, I. Senkovska, S. Kaskel, E. Brunner, *Langmuir* **2019**, *35*, 3162-3170.
